# Improve dosimetric outcome in stage III non-small-cell lung cancer treatment using spot-scanning proton arc (SPArc) therapy

**DOI:** 10.1186/s13014-018-0981-6

**Published:** 2018-02-27

**Authors:** Xiaoqiang Li, Peyman Kabolizadeh, Di Yan, An Qin, Jun Zhou, Ye Hong, Thomas Guerrero, Inga Grills, Craig Stevens, Xuanfeng Ding

**Affiliations:** Department of Radiation Oncology, Beaumont Health System, Royal Oak, MI USA

**Keywords:** Spot scanning, Proton arc therapy, Lung cancer, Robust treatment planning

## Abstract

**Background:**

To evaluate spot-scanning proton arc therapy (SPArc) and multi-field robust optimized intensity modulated proton therapy (RO-IMPT) in treating stage III non-small-cell lung cancer (NSCLC) patients.

**Methods:**

Two groups of stage IIIA or IIIB NSCLC patients (group 1: eight patients with tumor motion less than 5 mm; group 2: six patients with tumor motion equal to or more than 5 mm) were re-planned with SPArc and RO-IMPT. Both plans were generated using robust optimization to achieve an optimal coverage with 99% of internal target volume (ITV) receiving 66 Gy (RBE) in 33 fractions. The dosimetric results and plan robustness were compared for both groups. The interplay effect was evaluated based on the ITV coverage by single-fraction 4D dynamic dose. Total delivery time was simulated based on a full gantry rotation with energy-layer-switching-time (ELST) from 0.2 to 4 s. Statistical analysis was also evaluated via Wilcoxon signed rank test.

**Results:**

Both SPArc and RO-IMPT plans achieved similar robust target volume coverage for all patients, while SPArc significantly reduced the doses to critical structures as well as the interplay effect. Specifically, compared to RO-IMPT, SPArc reduced the average integral dose by 7.4% (*p* = 0.001), V_20_, and mean lung dose by an average of 3.2% (*p* = 0.001) and 1.6 Gy (RBE) (*p* = 0.001), the max dose to cord by 4.6 Gy (RBE) (*p* = 0.04), and the mean dose to heart and esophagus by 0.7 Gy (RBE) (*p* = 0.01) and 1.7 Gy (RBE) (*p* = 0.003) respectively. The average total estimated delivery time was 160.1 s, 213.8 s, 303.4 s, 840.8 s based on ELST of 0.2 s, 0.5 s, 1 s, and 4 s for SPArc plans, compared with the respective values of 182.0 s (*p* = 0.001), 207.9 s (*p* = 0.22), 250.9 s (*p* = 0.001), 509.4 s (*p* = 0.001) for RO-IMPT plans. Hence, SPArc plans could be clinically feasible when using a shorter ELST.

**Conclusions:**

This study has indicated that SPArc could further improve the dosimetric results in patients with locally advanced stage NSCLC and potentially be implemented into routine clinical practice.

## Background

Lung cancer remains the leading cause of cancer deaths for both men and women in the United States [1]. For patients with locally advanced lung cancer, radiotherapy alone or concurrent chemoradiotherapy has been widely used. Many trials have shown the benefits of concurrent chemoradiotherapy to improve the prognosis of patients, in terms of local control and survival [2–6]. However, the associated toxicities to the surrounding normal tissues can be significant, which could limit the ability of target dose escalation to possibly improve the outcome [7–12].

Proton beam therapy, unlike photon radiotherapy, has inherent physical characteristics which are advantageous to treat disease sites such as locally advanced lung cancer, due to its sharp distal dose fall-off. Compared to passive scatter proton therapy (PSPT) [13], intensity modulated proton therapy (IMPT) has shown potentials to further reduce the dose to the adjacent normal tissues while maintaining similar or superior target coverage [14–17] in a more efficient way without having the need to use beam specific blocks or compensators. In IMPT, thousands of mono-energetic narrow beamlets (“spots”) are optimized simultaneously and magnetically scanned to superpose a desired dose distribution. Although such active scanning delivery system provides the greatest flexibility to shape the target dose pattern, it can also be degraded by different uncertainties, including setup, range uncertainties [18–20], and the motion induced uncertainties [21–25].

Many studies have shown that robust optimization can effectively reduce the negative dosimetric impact from setup and range uncertainties compared to traditional margin approach in IMPT planning [26–32]. All studies on robust optimization, however, are still limited to few beam angles, due to low calculation speed and delivery efficiency. These obstacles limit our ability to further exploit the benefits of IMPT. With the recent development of spot-scanning arc therapy (SPArc) [33], which generates robust and delivery efficient spot scanning proton arc plans, we could potentially overcome the current dosimetric limitations [34, 35]. Herein, we evaluate the potential benefits of SPArc for treating locally advanced non-small-cell lung cancer (NSCLC) relative to the conventional multi-field robust optimized IMPT (RO-IMPT) plans.

## Methods

### Patient characteristics

Fourteen patients with stage IIIA or IIIB NSCLC (Table 1) treated with intensity modulated radiotherapy (IMRT) at our institution previously were selected for this study. All patients received four-dimensional (4D) CT simulation using a helical CT scanner (Philips Brilliance Big Bore, Philips Healthcare System, Cleveland, OH). The internal target volume (ITV) was defined on the average CT scan, which enveloped the gross target volumes (GTVs) on all individual respiratory phase CT scans. The ITV was then extracted with a 5 mm uniform expansion for clinical target volume (CTV). These fourteen patients were divided into two groups based on the extent of tumor motion. Group 1 contained eight patients with tumor motion less than 5 mm, while group 2 included six patients with tumor motion equal to or more than 5 mm. All the RO-IMPT plans were planned with three beams. The SPArc plans were generated using the SPArc algorithm with a partial arc starting from 10 degree initially with 2.5 degree sampling rate for the final plan. Both plans were optimized on average CT scan with the same following parameters: 3 mm uniform dose grid, 0.01 minimum monitor unit (MU) of spots, and ±3.5% range, 5 mm setup uncertainties (21 scenarios), to minimize the dose to adjacent critical normal structures with an adequate coverage of 99% ITV receiving 66 Gy (RBE) in 33 fractions. Similar objective constraints for organs at risks (OARs) were used for both plans.

**Table 1:** Patient Characteristics

Group	ID	Age	Location	TNM stage	Stage	ITV(cc)	Motion (cm)		
							SI	AP	LR
1	1	56	RUL	T3N2M0	IIIA	164.1	< 0.5		
	2	71	LUL	T1bN2M0	IIIA	107.0			
	3	75	LUL	T4N2M0	IIIB	822.8			
	4	49	RUL	T2aN2M0	IIIA	114.7			
	5	64	LLL	T3N2M0	IIIA	345.3			
	6	71	LLL	T2bN3M0	IIIB	265.4			
	7	51	LUL	T3N3M0	IIIB	366.5			
	8	56	RLL	T4N0M0	IIIA	276.7			
2	9	66	RLL + RML	T4N2M0	IIIB	350.6	0.5	0	0
	10	76	RML	T3N1M0	IIIA	157.1	0.7	0.1	0.1
	11	55	LLL	T1aN2M0	IIIA	734.6	0.6	0.1	0
	12	75	RUL	T2aN2M0	IIIA	77.4	0.5	0.2	0.1
	13	60	LLL	T2N2M0	IIIA	269.1	1.3	0.1	0.1
	14	50	RLL + RML	T4N2M0	IIIB	401.8	1.2	0.1	0.3

Abbreviations: *RUL* right upper lobe, *LUL* left upper lobe, *LLL* left lower lobe, *RLL* right lower lobe, *RML* right middle lobe

### SPArc algorithm

The SPArc algorithm was integrated into the worst-case scenario robust optimization in RayStation (version 5.0, RaySearch Laboratories AB, Stockholm, Sweden) to iteratively generate a proton arc plan with increasing control point sampling rate by the following steps: (1) control point re-sampling, (2) control point energy layers re-distribution, and (3) control point energy layers filtration. After the arc plan reaches a predefined control point sampling rate (e.g., 2–6 degrees), the SPArc algorithm continues (4) energy layers re-sampling and (5) spot number reduction to further assure the optimum solution while achieving the best treatment delivery efficiency. The details of such algorithm have been discussed by Ding et al. [33] .

### Plan evaluation

For plan quality evaluation, the dose volume histograms (DVHs) of both target volume and OARs were generated on nominal dose distributions. The following dosimetric indices were compared: the dose at which 99% (D_99%_) of the ITV volume was covered, the target maximum point dose (D_1%_), the percentages of the normalized volume of the total normal lung receiving more than 5 Gy, 10 Gy, 20 Gy, 30 Gy (V_5_, V_10_, V_20_, V_30_), the normal lung mean dose, the spinal cord maximum point dose (D_0.03cc_), the percentage of the normalized heart volume receiving more than 40 Gy (V_40_), the heart mean dose, and the esophagus mean dose. The integral dose (ID) with unit in joule was also calculated according to Murshed et al. [36] as the following:

1 $$ ID={\sum}_i{D}_i\ast {V}_i\ast {\rho}_i $$

where *D*_*i*_, *V*_*i*_, and *ρ*_*i*_ are the dose, voxel volume, and density values of the *i*th voxel in the body. The integral body dose describes the total energy imparted to the body.

The ITV coverage was further evaluated using the conformality index (CI), which was defined as [37]:

2 $$ CI=\frac{ TV Dp}{TV}\times \frac{ TV Dp}{VDp}, $$

where *TVDp*, *TV*, and *VDp* are the target volume covered by the prescribed dose, target volume, and the volume enclosed by the prescription isodose line respectively.

In terms of plan robustness quantification, both SPArc and RO-IMPT plans were re-calculated with 5 mm isocenter shift in the anterior-posterior, superior-inferior, and right-left directions under nominal proton beam range, with + 3.5% and − 3.5% proton beam ranges uncertainties, corresponding to total of 21 dose distribution scenarios. The DVHs for all the scenarios were plotted for comparison. The root-mean-square dose (RMSD) for each voxel was calculated, and the RMSD volume histograms (RVHs) and the areas under the RVH curve (AUC) [38] were computed for both target volume and OARs to compare the robustness. The smaller the AUC value was, the more robust the plan for the corresponding organ was.

For patients in group 2 with tumor motion equal to or more than 5 mm, the interplay effect were evaluated based on the average ITV coverage via the single-fraction 4D dynamic dose calculation without considering rescanning for different starting respiratory phases [23]. This 4D dynamic dose was calculated by synchronizing the breathing cycle with the machine delivery pattern [23, 25, 39] (e.g., assigning the spots on different respiratory phases of the CT simulation scan based on the time scale), and then accumulated via the deformable image registration on the expiration phase for evaluation, assuming the energy-layer-switching-time (ELST) of 1 s and a regular respiratory breathing period of 4.5 s.

The comparisons of treatment delivery efficiency between SPArc and RO-IMPT plans were simulated based on a full gantry rotation (360 degrees) with 1 rotation per minute rotation speed, 2 ms spot switching time, and ELST of 0.2 s, 0.5 s, 1 s, 2 s, and 4 s [33]. All the results were statistically analyzed with non-parametric Wilcoxon signed rank test using SPSS 19.0 software (International Business Machines, Armonk, New York), and *p* values equal to or less than 0.05 were considered statistically significant.

## Results

### Dosimetric improvement

**Fig. 1:**
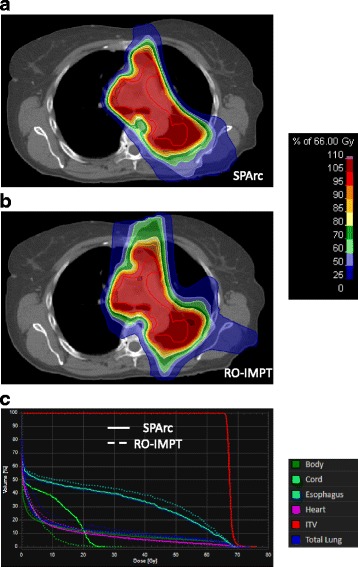
Dose distributions of (**a**) SPArc and (**b**) RO-IMPT plans, and the corresponding DVHs (solid line for SPArc, dashed line for RO-IMPT) comparisons for patient 6

Table 2 shows the average dosimetric parameters of fourteen patients comparing SPArc and RO-IMPT plans. With similar target coverage, in terms of D_99%_, D_1%_ dose to the target volume and conformality index, SPArc was able to significantly reduce the dose to all the critical structures evaluated. Specifically, SPArc plans decreased the average integral dose from 77.3 J to 72.0 J, by 7.4% (*p* = 0.001) compared to RO-IMPT plans. The average V_5_, V_10_, V_20_, V_30_ and mean lung dose for SPArc plans were 20.4%, 16.2%, 12.9%, 10.8%, and 7.9 Gy (RBE). While comparing to RO-IMPT plans, SPArc reduced the average V_5_, V_10_, V_20_, V_30,_ and mean lung dose by 4.6% (*p* = 0.001), 4.2% (*p* = 0.001), 3.2% (*p* = 0.001), 2.6% (*p* = 0.001), and 1.7 Gy (RBE) (*p* = 0.001) respectively. In terms of other OARs, SPArc reduced the max dose to cord by 4.6 Gy (RBE) (*p* = 0.04), the mean dose to heart and esophagus by 0.7 Gy (RBE) (*p* = 0.01) and 1.7 Gy (RBE) (*p* = 0.003) respectively compared to RO-IMPT plans. Figure 1 shows an example (patient 6) for both plans at nominal position.

**Table 2:** Average dosimetric results for the fourteen patients

Structures	Value	SPArc	RO-IMPT	*p* value
ITV	D_99%_(Gy)	66	66	N/A
	D_1%_(Gy)	70.9	71.1	0.22
Total Lung	V_5_(%)	20.4	25.0	0.001
	V_10_(%)	16.2	20.4	0.001
	V_20_(%)	12.9	16.1	0.001
	V_30_(%)	10.8	13.4	0.001
	Mean(Gy)	7.9	9.5	0.001
Cord	D_0.03cc_(Gy)	17.8	22.4	0.04
Heart	V_40_(%)	4.4	5.0	0.03
	Mean(Gy)	4.2	4.9	0.01
Esophagus	Mean(Gy)	12.9	14.6	0.003
ID	(J)	72.0	77.3	0.001
CI		0.70	0.68	0.33
DT (ELST 0.2 s)	(s)	160.1	182.0	0.001
DT (ELST 0.5 s)	(s)	213.8	207.9	0.22
DT (ELST 1 s)	(s)	303.4	250.9	0.001
DT (ELST 2 s)	(s)	482.5	337.1	0.001
DT (ELST 4 s)	(s)	840.8	509.4	0.001

Abbreviations: *ITV* internal target volume, *Cl* conformality index, *ID* integral dose, *DT* estimated delivery time, *ELST* energy layer switch time

### Robustness quantification

**Fig. 2:**
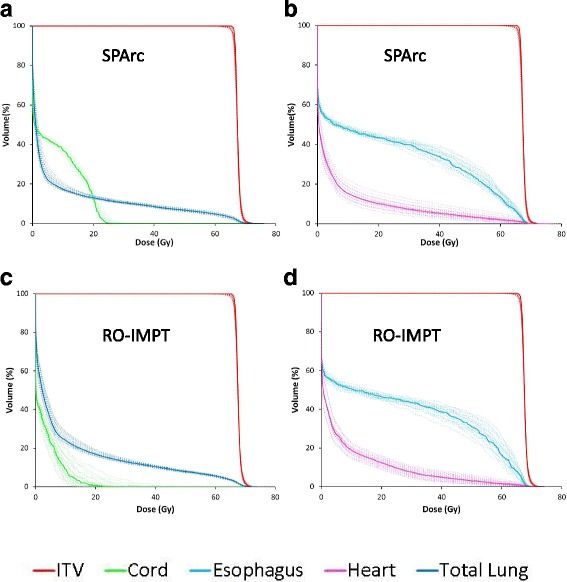
The dose distributions of (**a**, **b**) SPArc and (**c**, **d**) RO-IMPT plans for nominal position (solid line) and 20 scenarios of uncertainties (dashed line) for patient 6

**Fig. 3:**
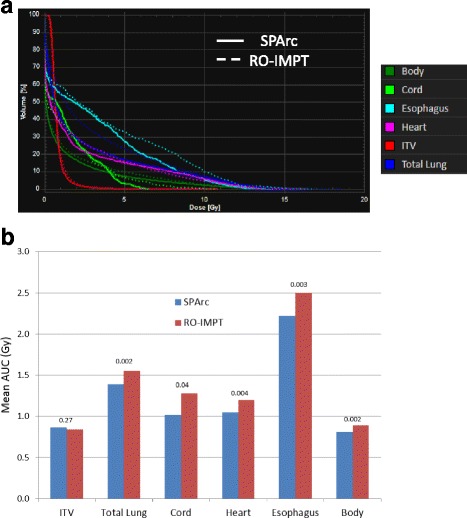
**a** The root-mean-square dose (RMSD) volume histograms of SPArc (solid line) and RO-IMPT (dashed line) for patient 6. **b** The average areas under the RVH curve (AUC) for fourteen patients (**b**) with *p*-values on the top of the columns

Figure 2 shows the DVHs for target volume and OARs in all 21 scenarios for SPArc and RO-IMPT plans for patient 6. Both plans could achieve an adequate coverage of at least 95% of ITV with the prescription dose under such uncertainties. Figure 3 shows the corresponding RVHs comparison for patient 6 (Fig. 3a) and the average mean AUC index of the fourteen cases and the corresponding *p* values (Fig. 3b). The average AUC index of the ITV, were very similar, with the corresponding average AUC values of 0.87 and 0.84 Gy (RBE) (*p* = 0.27) respectively for both plans. SPArc could significantly reduce the average AUC values from 1.55 Gy (RBE) to 1.39 Gy (RBE) (*p* = 0.002), 1.28 Gy (RBE) to 1.02 Gy (RBE) (*p* = 0.04), 1.20 Gy (RBE) to 1.05 Gy (RBE) (*p* = 0.004), 2.50 Gy (RBE) to 2.22 Gy (RBE) (*p* = 0.003), 0.89 Gy (RBE) to 0.81 Gy (RBE) (*p* = 0.002) for total normal lung, spinal cord, heart, esophagus, and integral body dose respectively.

### Delivery time

Table 2 lists the estimated delivery time per fraction for both SPArc and RO-IMPT plans for various ELST. For the proton centers with ELST equaling to 4 s, the estimated delivery time ratios between SPArc to RO-IMPT plans was 1.65 (840.8 s vs. 509.4 s), with the corresponding time significantly being longer (*p* = 0.001) for SPArc plans. As the ELST gets shorter to 0.5 s, the delivery time was still longer for SPArc plans but the differences were not statistically significant. With the ELST of 0.2 s, the SPArc plan could be delivered in a shorter time when compared to RO-IMPT (*p* = 0.001).

### The effect of target movement and interplay

**Fig. 4:**
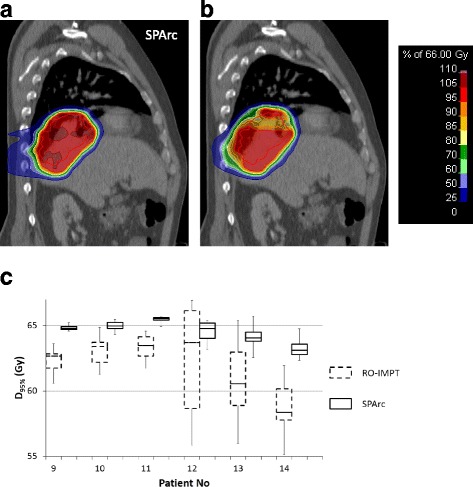
Single-fraction 4D dynamic dose distributions without considering rescanning on exhale phase for (**a**) SPArc and (**b**) RO-IMPT plan for patient 13 and the boxplot of the doses encompassing 95% (D_95%_) ITV on exhalation phase for group 2 patients based on single-fraction 4D dynamic dose simulated with different starting breathing phase (**c**)

SPArc has the potential to significantly reduce the interplay effect. More specifically, the average dose covering 95% of ITV via single-fraction 4D dynamic dose without considering rescanning was 64.5 Gy for SPArc compared to the value of 62.0 Gy (*p* = 0.01) for RO-IMPT. Figure 4a, b shows an example of 4D dynamic dose calculation of SPArc versus RO-IMPT plans for patient 13. In this evaluation, it is clearly noted that SPArc reduced the cold spots and hot spots induced by motion in the target volume. Figure 4c showed the boxplot of the doses encompassing 95% (D_95%_) of ITV on exhalation phase for group 2 patients with different starting breathing phase. The median and range of the D_95%_ are significantly improved via SPArc. Hence SPArc could effectively not only improve the target volume coverage but also decrease the extent of impact associated with respiratory phase treatment starting point on target coverage.

### The calculation speed of SPArc plan

The average calculation time required to generate a SPArc plan is 4 h, ranging from 3 to 6 h depending on the size of the target volume. This simulation was performed on a 64-bit workstation with an Intel Quad-Core processor (TM i5-4590 CPU @ 3.30 GHz) and 64 GB RAM.

## Discussion

This study evaluated the dosimetric benefits of using SPArc in treatment of stage III NSCLC patients. These results suggest that SPArc is able to significantly reduce the dose to all critical structures evaluated, while maintaining similar or superior target volume coverage and plan robustness compared to RO-IMPT plans. It is important to note that such additional organ dose sparing could potentially grant the clinician the ability to escalate the dose to the target volume safely. Although RTOG 0617 and a recent meta-analysis of randomized trials [40, 41] did not show a benefit of dose escalation in the chemoradiotherapy setting, the negative results could be partially related to high levels of toxicity, as dose escalation still appears to improve survival in the radiation alone setting. RTOG 0617 showed a strong association between overall survival and higher heart dose, especially the heart volume receiving more than 40 Gy [42]. Furthermore, the secondary analysis showed using IMRT planning also resulted in a significant reduction in grade 3 or higher pneumonitis. With techniques such as SPArc, shown here to further reduce lung and other normal tissues doses while concurrently reducing interplay effects related to respiratory tumor motion which may be critical in proton delivery, there may be further potential to dose escalate the target volume without increasing toxicity or mortality but potentially improving control or survival. As national cooperative groups compare proton to photon irradiation with survival endpoints, such as in the on-going RTOG 1308 and protons become more readily available in the community, these issues will become increasingly important.

**Fig. 5:**
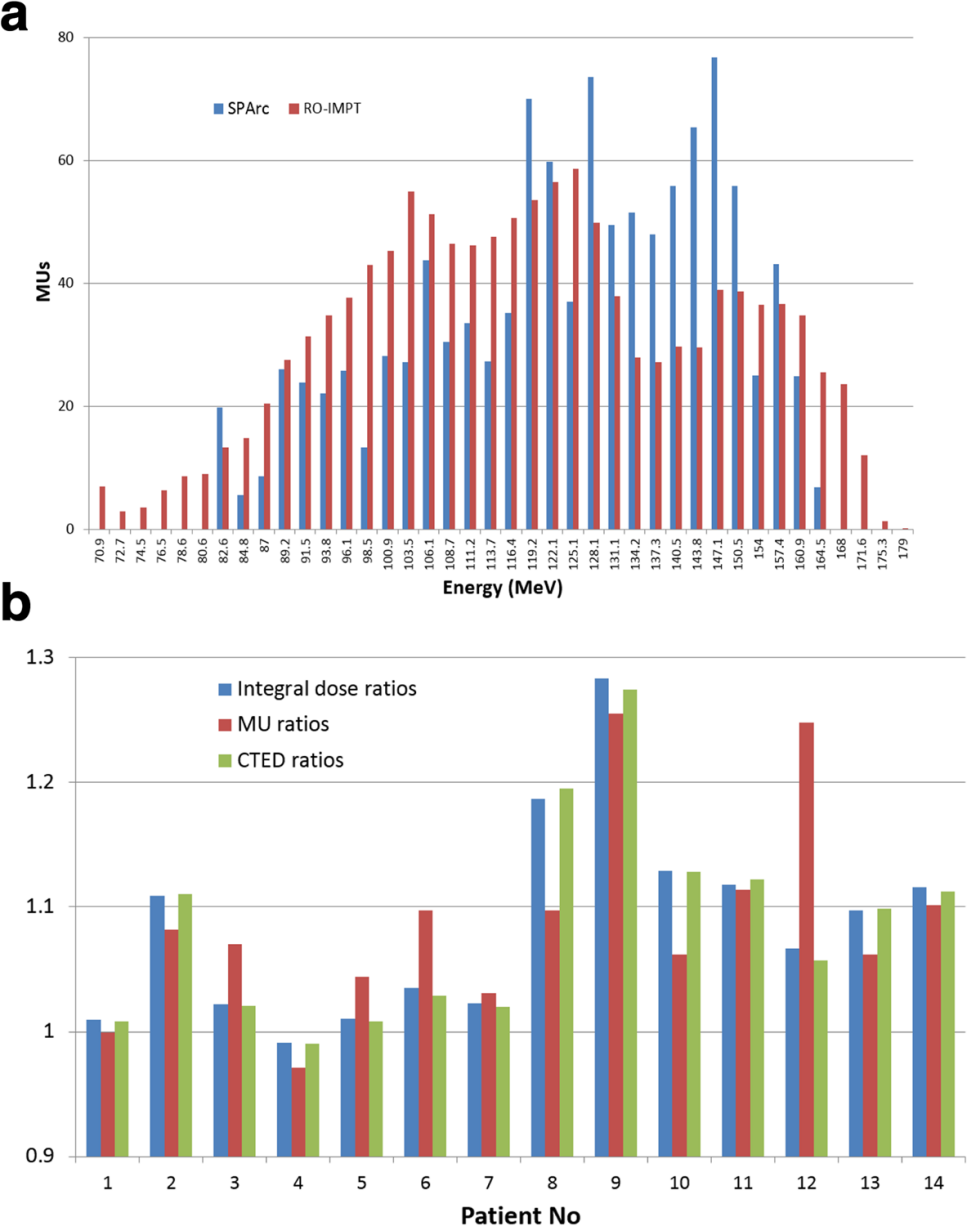
**a** The distribution of MUs and energies for all the beamlets used between SPArc and RO-IMPT for patient 6. **b** The integral dose, MU, and the calculated total energy depositions (CTED) ratios for the fourteen patients between RO-IMPT and SPArc plans

One counterintuitive result of our study is that SPArc plans could significantly decrease the integral body dose compared to RO-IMPT plans despite that SPArc generally uses hundreds of beams entering from different angles. One explanation is that the iteratively energy layer selection algorithm of SPArc optimization tends to filter the highest energy of each beam angle and superimpose the dose contributions from other beam angles to cover the distal extent of the target volume to produce a most robust plan. Figure 5a plots the MUs distributions versus energy of both SPArc and RO-IMPT plans for patient 6. As it is shown, the SPArc plan only used energies higher than 82.6 MeV and lower than 164.5 MeV while the RO-IMPT delivered a portion of the plan using energies higher than 164.5 MeV. In order to quantitatively illustrate this concept in more details, the calculated total energy depositions (CTED) were estimated as follows to evaluate the ratios between RO-IMPT and SPArc plans:

3 $$ CTED={\sum}_i MUi\ast Ei/S(Ei) $$

where *MUi*, *Ei*, *S*(*Ei*) are the monitor units, energy, and the corresponding proton stopping power for the energy in air for *i*th energy layer. Such CTED ratios roughly estimate the total energy depositions between these two modalities. Figure 5b shows the ratios among integral dose, total MUs, and CTED. The integral dose ratios match very well with the CTED ratios, which ultimately explain the reduction of integral body dose for the SPArc plan.

One of the major challenges with scanning beam proton therapy in treatment of lung cancer patients is the interplay effect between the spot scanning delivering sequence and the patient specific respiratory motion. Such interplay effect could lead to severe dose distortion especially in cases of large tumor motion, and could possibly be diminished by rescanning [23, 25, 43, 44]. In this study for a fair comparison, we only compared the single-fraction 4D dynamic dose calculation without taking rescanning into account. The results demonstrated the ability of SPArc to reduce the interplay effect as shown in Fig. 4. This improvement could be explained by the washout effect of SPArc’s hundreds of beam angles. Further studies are needed, however, evaluating different tumor motions and ELST simulating different patients and machine parameters to evaluate the interplay effect and SPArc more comprehensively. In routine clinical practice of using IMPT for motion target, rescanning technique is normally used in order to mitigate such interplay effects.

Moreover, it is important to mention that another potential benefit of using SPArc over the RO-IMPT plan could be the relative biological effectiveness (RBE) optimization. The RBE of 1.1 has been used historically and conservatively to ensure target coverage without taking into account the variation of RBE along the beam path. Paganetti et al. [45] has systemically analyzed the published experimental data and supported that there is an increase of RBE to 1.7 towards the distal falloff of the spread-out Bragg peak. Unlike traditional passive scatter proton therapy, IMPT has the potential to intentionally place the higher RBE around the Bragg peak inside the target volume to avoid any extra biological damage to the surrounding normal tissue. Furthermore, in case of SPArc, using hundreds of beam angles along with great energy layer selection magnetism would provide an even greater flexibility to locate the high RBE points inside the target volume. As indicated in Fig. 5a, even without biological optimization, the SPArc plan tends to avoid using very low and high energy beams, and therefore allows a greater probability of placing Bragg Peak into the target volume.

SPArc, as demonstrated here, shows significant potential to further improve the dosimetric outcome and therapeutic ratio in treating locally advanced NSCLC compared to RO-IMPT. Due to the limitations of current proton scanning beam systems, continuous gantry rotation with scanning delivery is still not available. Issues that need to be addressed in order to efficiently and safely implement SPArc for routine clinical use are: (1) developing a new rotational gantry which is capable of rotating around the iso-center while delivering pencil beam therapy with sub-millimeter accuracy; (2) designing new beamline control system to adjust and monitor the beam energy spectrum, spot position and spot profile during the gantry rotation, and (3) implementing a comprehensive SPArc quality assurance program.

## Conclusions

SPArc is a robust and delivery-efficient proton spot-scanning arc therapy technique which could potentially be implemented into routine clinical practice to further improve treatment outcomes in patients with locally advanced NSCLC.

## Abbreviations

AUC: Area Under the RVH curve
CI: Conformality Index
CTED: Calculated total energy depositions
CTV: Clinical Target Volume
D_0.03cc_: Dose received by 0.03cc volume
D_1%_: Dose received by 1% of volume
D_95%_: Dose received by 95% of volume
D_99%_: Dose received by 99% of volume
DVHs: Dose Volume Histograms
ELST: Energy Layer Switching Time
GTV: Gross Target Volume
ID: Integral Dose
IMPT: Intensity Modulated Proton Therapy
IMRT: Intensity Modulated Radiation Therapy
ITV: Internal Target Volume
MU: Monitor Unit
NSCLC: non-small-cell lung cancer
OARs: Organ-at-risks
PSPT: Passive Scatter Proton Therapy
RBE: Relative Biological Effectiveness
RMSD: Root-mean-square dose
RO-IMPT: Robust Optimized Intensity Modulated Proton Therapy
RVHs: RMSD volume histograms
SPArc: Spot-Scanning Proton Arc
V5: Volume received at least 5Gy
